# The efficacy and safety of silodosin for the treatment of ureteral stones: a systematic review and meta-analysis

**DOI:** 10.1186/s12894-016-0141-y

**Published:** 2016-05-27

**Authors:** Diandong Yang, Jitao Wu, Hejia Yuan, Yuanshan Cui

**Affiliations:** Department of Urology, Yantai Yuhuangding Hospital Affiliated to Medical College of Qingdao University, NO.20 East Yuhuangding Road, 264000 Yantai, China

**Keywords:** Silodosin, Ureteral stones, Meta-analysis, Randomized controlled trial

## Abstract

**Background:**

To evaluate the efficacy and safety of silodosin as a medical expulsive therapy for ureteral stones by means of a systematic review and meta-analysis.

**Methods:**

We searched MEDLINE, EMBASE and the Cochrane Controlled Trials Register to identify randomized controlled trials (RCTs) of silodosin in the treatment of ureteral stones. The reference lists of retrieved studies were also investigated.

**Results:**

Six RCTs, including 916 participants and comparing silodosin with controls, were used in the meta-analysis. Silodosin was superior to controls in terms of stone expulsion rate, the primary efficacy end point in all six RCTs (odds ratio [OR] for expulsion 2.16, 95 % confidence interval [CI] 1.62 to 2.86, *p* <0.00001). Silodosin was also more effective for secondary efficacy end points; the stone expulsion time (standardized mean difference [SMD] −3.66, 95 % CI −6.61 to −0.71; *p* =0.01) and analgesic requirements (SMD −0.89, 95 % CI −1.19 to −0.60; *p* < 0.00001) were significantly reduced compared with those of controls. Other than the incidence of abnormal ejaculation, which was higher in the silodosin groups (OR 2.84, 95 % CI 1.56 to 5.16, *p* =0.0006), few adverse effects were observed.

**Conclusion:**

This meta-analysis indicates silodosin is an effective and safe treatment option for ureteral stones with a low occurrence of side effects.

## Background

Urolithiasis is a multifactorial disease that is common in daily urological practice, and is also a substantial public health problem. After urinary tract infections and pathologic conditions of the prostate [1], urolithiasis is the third most common disease of the urinary tract, with an estimated prevalence of 2–3 % and a lifetime recurrence rate of approximately 50 % [2, 3]. To date, minimally invasive therapies, such as extracorporeal shock wave lithotripsy, ureterolithotripsy and percutaneous nephrolithotomy have proved to be effective treatments in many cases. Nevertheless, these procedures are expensive and are not without risk [4]. A conservative approach involving close monitoring can be used in most cases, and is becoming more popular as a result of advances in pharmacological therapy, which can reduce symptoms and facilitate stone expulsion [5, 6]. For example, medical expulsive therapy (MET) using α-adrenoceptor antagonists has emerged as an alternative strategy for the initial management of small distal ureteral stones [7].

Silodosin is a novel highly selective α1A-adrenoceptor (α1A-AR) blocker: *in vitro* its α1A-to-1B binding ratio is extremely high (162:1), suggesting that it has the potential to reduce dynamic neurally mediated smooth muscle relaxation in the ureter, while minimizing undesirable effects on blood pressure regulation [8].

The goal of this study was to perform a meta-analysis to evaluate the efficacy and safety of silodosin as a MET for ureteral stones to help address some of the current controversies over its use for this indication.

## Methods

### Search strategy

MEDLINE (1966 to Jan 2015), EMBASE (1974 to Jan 2015) and the Cochrane Controlled Trials Register databases were searched to identify randomized controlled trials (RCTs) of silodosin in the treatment of ureteral stones; we also searched the reference lists of the retrieved studies. The following search terms were used: “silodosin”; “ureteral stones”; and “randomized controlled trial”.

### Inclusion criteria and trial selection

**Fig. 1:**
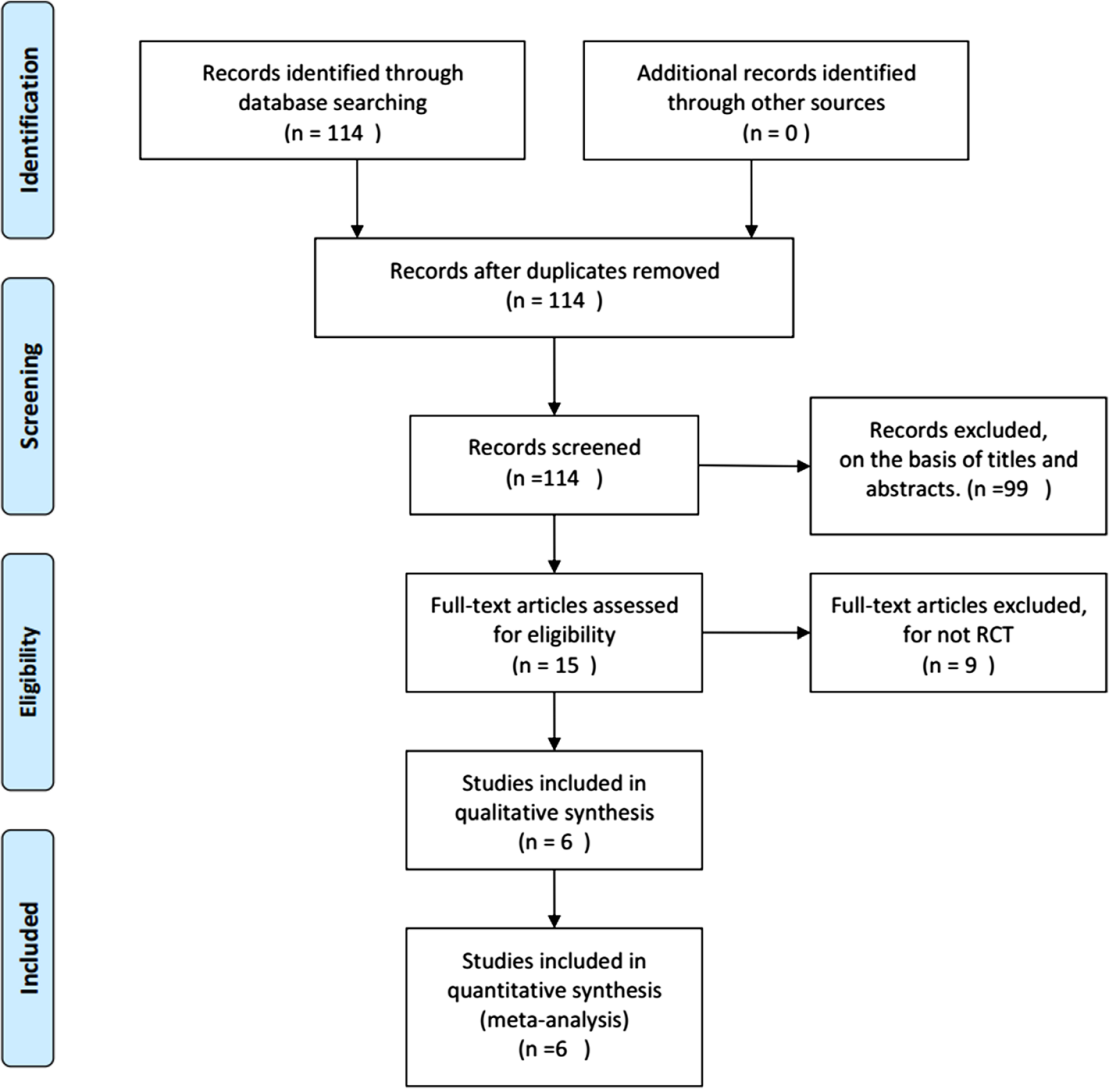
A flow diagram of the study selection process. RCT: randomized controlled trial

Randomized controlled trials that met the following criteria were included: (1) the study design included treatment with silodosin; (2) the study provided accurate data that could be analyzed, including the total number of subjects and the values of each outcome measured; and (3) the full text of the study could be accessed. When the same study was published in more than one journal or in different years, the most recent publication was used for the meta-analysis. If the same group of researchers studied a group of subjects with multiple experiments, then each study was included. A flow diagram of the study selection process is presented in Fig. 1.

### Quality assessment

The quality of the retrieved RCTs was assessed using the Jadad scale [9]. All identified RCTs were included in the meta-analysis, regardless of the quality score. The methodological quality of each study was assessed according to the means of allocation of participants to the arms of the study, the concealment of allocation procedures, blinding and data loss due to attrition. The studies were then classified qualitatively according to the guidelines published in the *Cochrane Handbook for Systematic Reviews of Interventions* v.5.1.0 [10]. Each study was rated according to these quality assessment criteria, and assigned to one of the three following quality categories: A, if all quality criteria were adequately met the study was deemed to have a low risk of bias; B, if one or more of the quality criteria was only partially met or was unclear the study was deemed to have a moderate risk of bias; or C, if one or more of the criteria was not met or not included the study was deemed to have a high risk of bias. Differences were resolved by discussion among the authors.

### Data extraction

The following information was collected for each study: (1) the name of the RCT; (2) the study design and sample size; (3) the therapy that the patients received; (4) the country in which the study was conducted; and (5) data including the stone expulsion rate, stone expulsion time, analgesics required and incidence of adverse events, including abnormal ejaculation in male participants.

### Statistical analysis and meta-analysis

The meta-analysis of comparable data was carried out using RevMan v.5.1.0 (Cochrane Collaboration, Oxford, UK) [10]. We estimated the relative risk for dichotomous outcomes and the standardized mean difference (SMD) for continuous outcomes pooled across studies by using the DerSimonian and Laird random-effects model [11]. The corresponding 95 % confidence interval (CI) was calculated, if the result of analysis showed *p* >0.05, we considered the studies homogeneous and so chose a fixed-effect model for meta-analysis; otherwise, a random-effect model was used. We quantified inconsistency using the *I*^*2*^ statistic, which describes the proportion of heterogeneity across studies that is not due to chance, thus describing the extent of true inconsistency in results across trials [12]. *I*^*2*^ < 25 % reflects a small amount of inconsistency and *I*^*2*^ > 50 % reflects significant inconsistency.

## Results

### Characteristics of the individual studies

The database search produced 114 articles that could have been included in our meta-analysis. Based on the inclusion and exclusion criteria, 99 articles were excluded after reading the titles and abstracts of the articles; nine articles were not RCTs. In all, six articles [13–18], reporting data from three RCTs that compared silodosin with tamsulosin, two RCTs that compared silodosin with placebo and one RCT that compared silodosin with naftopidil were included in the analysis (Fig. 1). The baseline characteristics of the studies included in our meta-analysis are listed in Table 1.

**Table 1:** Study and patient characteristics

Study	Therapy in experimental group	Therapy in control group	Country	Sample size		Administration method	Duration of treatment	Dosage	Stone location and size range
				experimental	Control				
Itoh Y 2011	silodosin	blank control	Japan	95	92	Oral	8 wk	8mg/d	symptomatic unilateral ureteral calculi of less than 10 mm
Tsuzaka Y 2011	silodosin	naftopidil	Japan	35	39	Oral	6 wk	8mg/d	symptomatic≤10 mm ureteral stones
Guptas S 2013	silodosin	tamsulosin	India	50	50	Oral	4 wk	8mg/d	unilateral, uncomplicated middle or lower ureteral stones ≤10 mm
Dell'Atti L 2014	silodosin	tamsulosin	Italy	68	68	Oral	3 wk	8mg/d	single, unilateral, radiopaque, proximal ureteral stone (range 4–10 mm in size)
Sur RL 2014	silodosin	placebo	USA	119	120	Oral	4 wk	8mg/d	unilateral ureteral calculus of 4–10 mm
Kumar S 2015	silodosin	tamsulosin	India	90	90	Oral	4 wk	8mg/d	distal ureteric stones of size 5–10 mm

### Quality of the individual studies

**Fig. 2:**
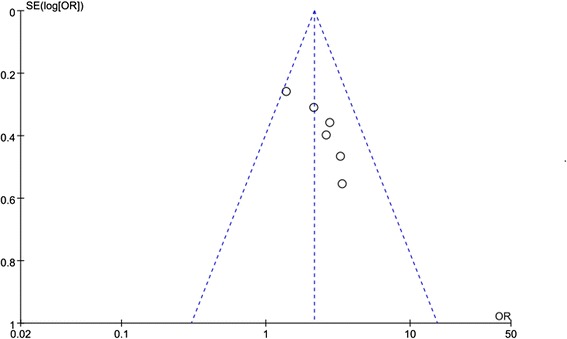
Funnel plot of the studies represented in our meta-analysis. OR: odds ratio, SE: standard error

Two of the six RCTs were double-blinded, and included descriptions of the randomization processes used. Three RCTs included a power calculation to determine the optimal sample size (Table 2). The quality of all identified studies was categorized as A or B, and the final Jadad score for each study ranged from 3 to 5 points (Table 2). A funnel plot suggested there was no bias (Fig. 2).

**Table 2:** Quality assessment of individual study

Study	Allocation sequence generation	Allocation concealment	Blinding	Loss to follow-up	Calculation of sample size	Statistical analysis	Level of quality	Jadad Score(5-point)
Itoh Y 2011	B	B	A	6	NO	Student’s t-test	B	3
Tsuzaka Y 2011	B	B	A	10	NO	Student’s t-test	B	3
Guptas S 2013	A	B	A	0	NO	Student’s t-test	A	4
Dell'Atti L 2014	B	A	A	3	YES	Student’s t-test	A	4
Sur RL 2014	A	A	A	6	YES	Wilcoxon test	A	5
Kumar S 2015	A	A	A	6	YES	chi-square test	A	5

A - all quality criteria met (adequate): low risk of bias. B - one or more of the quality criteria only partly met (unclear): moderate risk of bias

C - one or more criteria not met (inadequate or not used): high risk of bias

### Efficacy

#### Stone expulsion rate

**Fig. 3:**
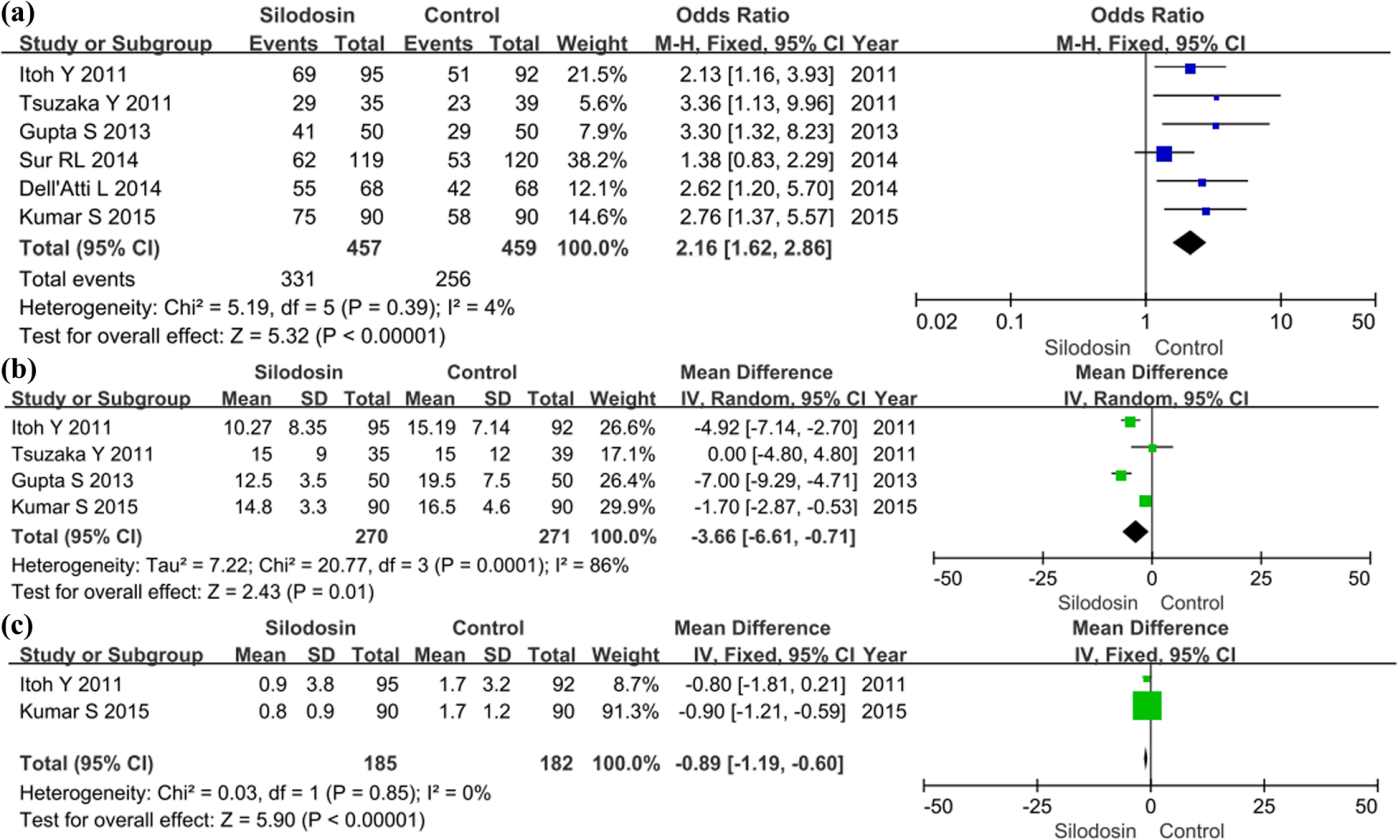
Forest plots showing changes in (**a**) the stone expulsion rate, (**b**) stone expulsion time and (**c**) analgesics were required. MH: mantel haenszel, CI: confidence interval, SD: standard deviation, IV: inverse variance

Six RCTs with 916 participants (457 in the silodosin groups and 459 in the control groups, Fig. 3) reported stone expulsion rate as the primary outcome measure. According to our analysis, no heterogeneity was found between the trials (*P* = 0.39) (Fig. 3), and a fixed-effects model was thus chosen for the analysis. Silodosin showed a significantly superior stone expulsion rate compared with controls (OR 2.16, 95 % CI 1.62 to 2.86; *p* <0.00001).

#### Stone expulsion time

Four RCTs with 541 participants (270 in the silodosin groups and 271 in the control groups, Fig. 3) reported stone expulsion times as a secondary outcome. According to our analysis, heterogeneity was found between the trials (*P* = 0.0001). A random-effects model was chosen for the analysis. The stone expulsion time was significantly shorter in the silodosin groups than controls (SMD −3.66, 95 % CI −6.61 to −0.71; *p* =0.01).

#### Analgesia required

Two of the RCTs (consisting of 367 participants, with 185 in the silodosin groups and 182 in the control groups, Fig. 3) reported the analgesics required during stone expulsion. According to our analysis, no heterogeneity was found between the trials (*P* = 0.85). A fixed-effects model was chosen for the analysis. Expulsion using silodosin was associated with a significantly lower analgesic requirement than that in controls (SMD −0.89, 95 % CI −1.19 to −0.69; *p* <0.00001).

### Side effects and safety

#### Abnormal ejaculation

**Fig. 4:**
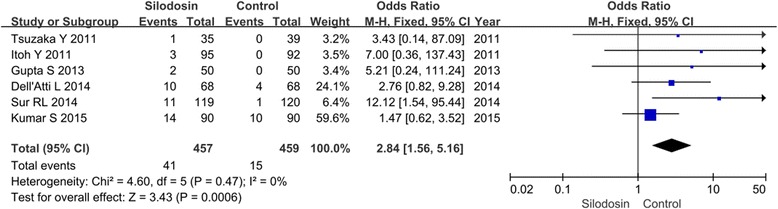
Forest plots showing changes in abnormal ejaculation. MH: mantel haenszel, CI: confidence interval

Six RCTs with 916 participants (457 in the silodosin groups and 458 in the control groups, Fig. 3) reported the incidence of abnormal ejaculation (Fig. 4). The effect size for the purposes of meta-analysis was denoted as the OR. According to our analysis based on a fixed-effects model, no heterogeneity was found between the trials (*P* = 0.47). The pooled estimate of OR was 2.84 (95 % CI 1.56 to 5.16, *p* =0.0006). This suggests that abnormal ejaculation was more common among patients treated with silodosin than among control-treated patients.

### Subgroup analysis

**Fig. 5:**
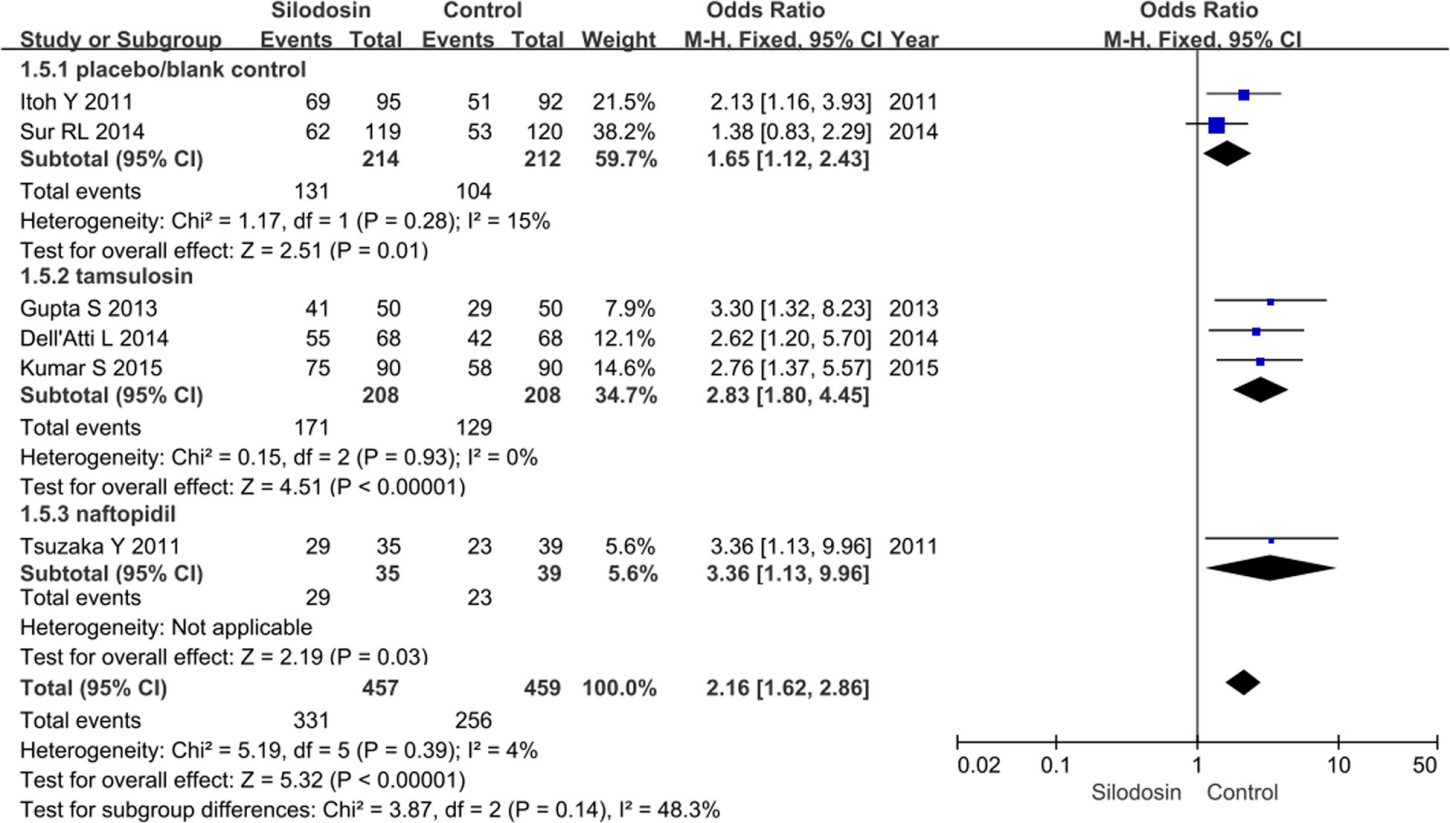
Forest plots showing changes in the stone expulsion rate. MH: mantel haenszel, CI: confidence interval (Subgroup analysis results)

We divided the included studies into three groups on the basis of the treatment given to the control groups: tamsulosin, naftopidil or inactive placebo. According to our analysis, no heterogeneity was found between the trials (*P* > 0.05); therefore, we chose a fixed-effects model for the analysis. Stone expulsion rates were significantly higher in those treated with silodosin compared with tamsulosin (OR 1.65, 95 % CI 1.12 to 2.43; *p* =0.01), naftopidil (OR 2.83, 95 % CI 1.80 to 4.45; *p* <0.00001) or inactive placebo (OR 3.36, 95 % CI 1.13 to 9.96; *p* =0.03, Fig. 5).

**Fig. 6:**
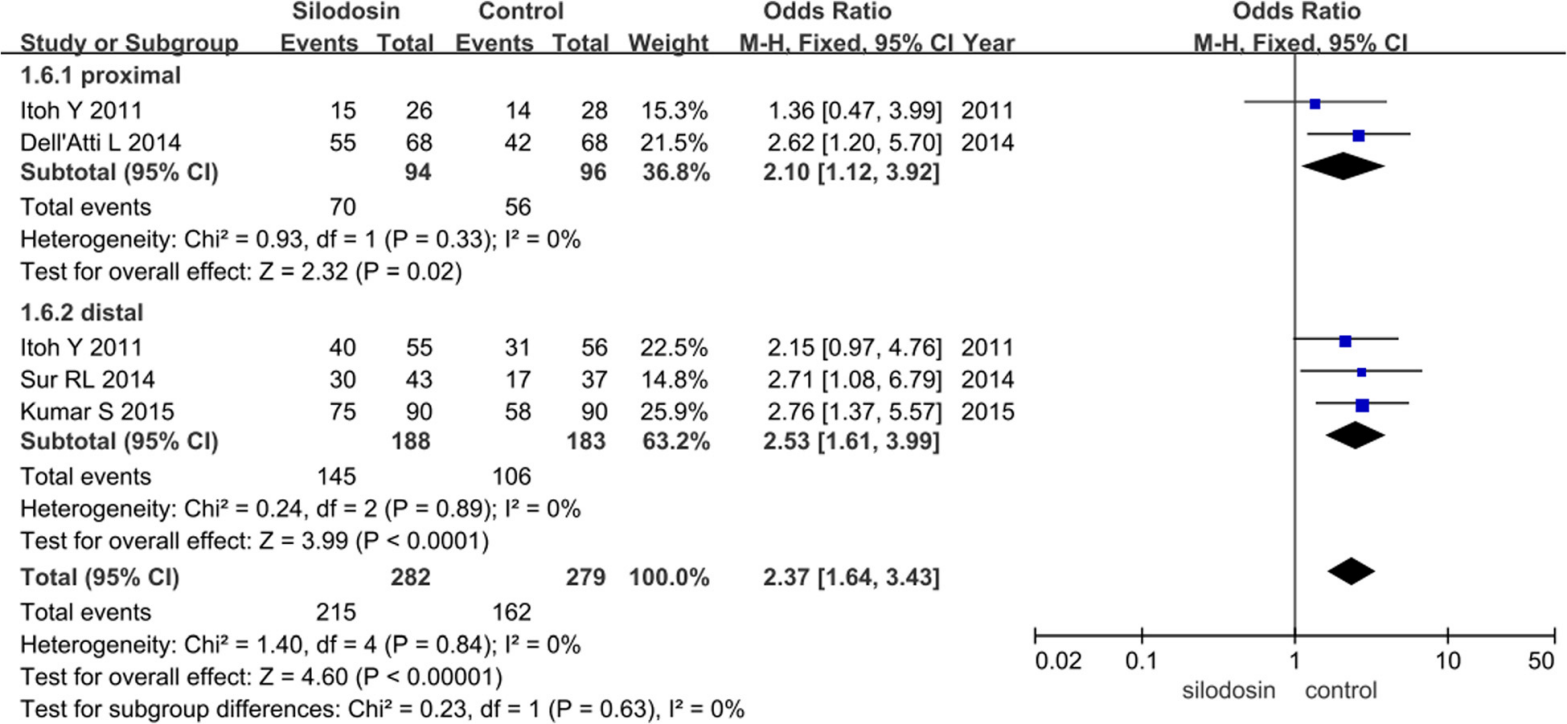
Forest plots showing changes in the stone expulsion rate. MH: mantel haenszel, CI: confidence interval (Subgroup analysis results)

We further divided the included studies into two groups according to stone location: proximal or distal. According to our analysis, no heterogeneity was found between the trials (*P* > 0.05); therefore, a fixed-effects model was chosen. Stone expulsion rates were significantly higher in those treated with silodosin compared with control in spite of the stone location. (proximal OR 2.1, 95 % CI 1.12 to 3.92; *p* =0.02 or distal OR 2.53, 95 % CI 1.61 to 3.99; *p* <0.00001, Fig. 6).

## Discussion

The introduction of more effective drugs has seen significant improvements in the medical management of ureteral stones. The likelihood of a ureteral stone passing depends on several factors, which include the stone size and location, and the condition of the ureter [19]. The stimulation of the α1-AR in the ureter increases the force of ureteric contraction and the frequency of ureteric peristalsis. Blockade of the α1-AR inhibits basal tone, reduces peristaltic amplitude and frequency, and decreases intraluminal pressure while increasing the rate of fluid transport and the chances of stone expulsion. Expression of the α1A- and α1D-AR subtypes is greater in the distal ureter [20]. Silodosin is a highly selective α1A-AR blocker, and it has been demonstrated *in vitro* that silodosin’s α1A-to-1B binding ratio is extremely high (162:1).

Our meta-analysis found that silodosin 8 mg/day for 3–8 weeks is superior to controls (tamsulosin 0.4 mg/day, naftopidil 50 mg/day or inactive placebo) in improving the stone expulsion rate, reducing the stone expulsion time and analgesic requirements. According to our analysis, no heterogeneity was found between the trials, allowing us to use a fixed-effects model for the analysis. We may therefore conclude that silodosin 8 mg/day treats stones more effectively than tamsulosin 0.4 mg/day, naftopidil 50 mg/day or placebo. Our subgroup analysis showed that stone expulsion rates were significantly higher in those treated with silodosin compared with those treated with a control, regardless of stone location. It is worth noting that stone expulsion rates ranged from 57.7 % to 80.9 % in cases involving proximal ureteral stones, and 69.8 % to 93.8 % in distal ureteral stones. Therefore, it seems that the stone expulsion rate for distal ureteral stones is higher than for proximal ureteral stones. Stone size has been identified as an important predictive factor for expulsion: the trials we chose examined those ≤10 mm in diameter, so we cannot draw any conclusions about the role of silodosin in the treatment of larger ureteral calculi.

The α1A/D selective AR-blocker tamsulosin is recognized as a safe and effective drug that also enhances spontaneous passage of distal ureteral stones ≤10 mm in diameter [21]. Recent studies have demonstrated that the α1A subtype plays the most important role in mediating phenylephrine-induced contraction of the isolated human ureter [22]. Kobayashi et al. found that silodosin enhanced noradrenaline-induced contraction of the human ureter more than the selective α1D-AR antagonist BMY-7378 [23]. The mechanism of action of silodosin presumably includes blockade of the α-adrenergic receptors, thereby relaxing the ureter and potentially providing a spasmolytic effect [24].

Our meta-analysis suggests that there is a higher incidence of retrograde ejaculation in patients treated with silodosin than active or inactive controls. The incidence of side effects was similar to that reported by other authors [25]. Nonetheless, retrograde ejaculation does not appear to be particularly troublesome, and only a small proportion of participants enrolled in clinical studies who report this adverse effect discontinued treatments because of it [26]. Furthermore, retrograde ejaculation resolves completely within a few days of discontinuing treatment [26]. Silodosin appears to relax the smooth muscles of the lower urinary tract and the genital tract enough to induce retrograde ejaculation, reflected in the finding that patients who had the greatest relief from lower urinary tract symptoms had a higher likelihood of retrograde ejaculation. This observation suggests that retrograde ejaculation is an indirect indicator of the extent of the smooth muscle relaxation that silodosin induces. Other than retrograde ejaculation, the type and incidence of adverse events reported by those taking silodosin were similar to those taking tamsulosin, naftopidil or an inactive control. Besides, Imperatore V et al. conducted a retrospectively controlled study demonstrated that MET with silodosin is associated with a lower incidence of side effects related to peripheral vasodilation but an higher incidence of retrograde ejaculation when compared to tamsulosin [27].

Our meta-analysis was based on data collected entirely from RCTs that we considered to be at low risk of bias. This suggests that our findings could be sufficiently sound to inform everyday clinical practice. Importantly, however, the number of included studies was small and there were a variety of control groups; therefore, a certain amount of clinical heterogeneity seems inevitable. Furthermore, we cannot account for the possible influence of unpublished studies, which could have introduced an unrecognized bias into our analysis. The longer-term efficacy and safety of silodosin cannot therefore be extrapolated from our findings. More high-quality trials with larger sample sizes are needed to establish fully the role of silodosin in the treatment of distal ureteral stones.

## Conclusions

This meta-analysis indicates silodosin is an effective and safe treatment option for ureteral stones with a low occurrence of side effects.

## Abbrievations

RCT: Randomized controlled trial
OR: Odds ratio
CI: Confidence interval
SMD: Standardized mean difference
MET: Medical expulsive therapy
α1A-AR: α1A-adrenoceptor
